# Assessment of *Aedes albopictus* reference genes for quantitative PCR at different stages of development

**DOI:** 10.1371/journal.pone.0194664

**Published:** 2018-03-19

**Authors:** Najat Dzaki, Ghows Azzam

**Affiliations:** 1 School of Biological Sciences, Universiti Sains Malaysia, Penang, Malaysia; 2 Vector Control and Research Unit, School of Biological Sciences, Universiti Sains Malaysia, Penang, Malaysia; Centro de Pesquisas René Rachou, BRAZIL

## Abstract

Members of the *Aedes* genus of mosquitoes are widely recognized as vectors of viral diseases. *Ae*.*albopictus* is its most invasive species, and are known to carry viruses such as Dengue, Chikugunya and Zika. Its emerging importance puts *Ae*.*albopictus* on the forefront of genetic interaction and evolution studies. However, a panel of suitable reference genes specific for this insect is as of now undescribed. Nine reference genes, namely *ACT*, *eEF1-γ*, *eIF2α*, *PP2A*, *RPL32*, *RPS17*, *PGK1*, *ILK* and *STK* were evaluated. Expression patterns of the candidate reference genes were observed in a total of seventeen sample types, separated by stage of development and age. Gene stability was inferred from obtained quantification data through three widely cited evaluation algorithms i.e. BestKeeper, geNorm, and NormFinder. No single gene showed a satisfactory degree of stability throughout all developmental stages. Therefore, we propose combinations of *PGK* and *ILK* for early embryos; *RPL32* and *RPS17* for late embryos, all four larval instars, and pupae samples; *eEF1-γ* with *STK* for adult males; *eEF1-γ* with *RPS17* for non-blood fed females; and *eEF1-γ* with *eIF2α* for both blood-fed females and cell culture. The results from this study should be able to provide a more informed selection of normalizing genes during qPCR in *Ae*.*albopictus*.

## Introduction

*Aedes albopictus* (*Ae*. *albopictus*) is the most widely-travelled member of the *Aedes* genus [1]. Due to increased trade and ease of transcontinental travel [2,3], they are now found abundantly in not only the tropical Asian countries from which they originate, but also temperate Asia, Europe, the Americas, Australia, and Africa [4]. Though less notorious than *Aedes aegypti (Ae*. *Aegypti)* as a vector of arboviruses, *Ae*. *albopictus* is acknowledged as an efficient vector of at least 22 viral strains [5]. It also carries a multitude of cross-species infecting bacteria [6,7]. Throughout the course of history, this mosquito has been deemed responsible for a number of major outbreaks of diseases normally related to *Ae*. *aegypti*, such as Dengue [8,9] and Chikugunya [10]. *Ae*. *albopictus* is additionally susceptible to Zika, with high dissemination and transmission rates [11]. Nonetheless, it is typically presumed that where *Ae*. *aegypti* populations are rampant, *Ae*. *albopictus* would assume a secondary role to the former where the propagation of a virus is concerned. The geographical overlap and subsequent competitiveness between the two species is in fact often considered a strategy for *Ae*. *aegypti* control. However, *Ae*. *albopictus* appears to be more acclimatisable, and in more recent times has displaced *Ae*. *aegypti* as the dominant species in various locales [12]. As reports of a particular strain of Chikugunya evolving to be more transmissible by *Ae*. *albopictus* have emerged [13], worries regarding the possibility of other arboviruses also exhibiting such plasticity with regards to transmissibility by *Ae*. *albopictus* has grown and thus, understanding the host-virus interactions within the species as well as its efficiency as a propagator are areas of study gaining much interest.

An influx of gene expression studies is thus anticipated in the near future. Since it was first introduced in the 1990s, quantitative real-time polymerase chain reaction (qPCR) has become the main technique of choice for such purposes. This method requires minimal nucleic acid quantities compared to more its more traditional RNA-quantification compatriots, in addition to bearing the added advantages of being faster and more reproducible [14]. Its vast popularity has also made ready a variety of commercially accessible reagents and automated platforms. However, inconsistencies in protocol, template quality, as well as enzymatic efficiencies brought forth by such diverse product-availability have contributed to irregularities in qPCR data interpretation [15,16]. Normalization of data against a reference gene is therefore critical. This involves comparing the ratios of expression levels of the target gene against that of the selected reference gene(s) [17]. The importance of such an exercise is twofold; normalization not only compensates for variations in starting cDNA quantities amongst samples, but also lends the significance in differences seen from quantification data a higher degree of confidence [18,19]. Reference genes such as *TUBB* and *GAPDH* are examples of genes utilized heavily for this purpose. The commonality of this practice is borne out of the assumption that a reference gene is expressed consistently throughout the cell cycle. However, this is no longer considered true. No one gene has been found to be stably expressed under all developmental and experimental conditions [20–23]. A reference gene may in fact be specific towards a protocol and/or biological sample. Algorithms such as BestKeeper, geNorm, and NormFinder have since been developed to identify the best-fit reference gene to use with consideration to one’s unique experimental variables [17,24].

In *Ae*. *albopictus*, *ACT* [25] or *RPS7* [26] are often applied as the normalizing reference gene. However, the application of a solitary gene for this task has been dismissed by many as a flawed approach. The gene validation algorithms as aforementioned oftentimes recommend that at the very least, a combination of two candidates should be used together to achieve proper normalization [27–29]. This is especially crucial for organisms subjected to a wide array of environmental changes throughout its development. Like *Ae*. *aegypti*, *Ae*. *albopictus* is a holometabolous organism undergoing complete metamorphosis, and spends half its life-cycle as an aquatic organism. Minute diet and temperature differences are known to affect growth and development patterns of larvae and adult *Ae*. *aegypti* [30–32]. Expression levels of certain genes in female adults of both *Ae*. *aegypti* and *Ae*. *albopictus* have also been shown to depend on whether the individual has partaken a blood-meal [33,34]. As a number of these genes do appear to affect the mother-offspring transmission of viruses as well as the ability of the mosquito to carry viruses as adults, there is thus a need for the stability of candidate reference genes to be validated at different points of development to ensure robustness in gene expression normalization for this pathologically important species.

Here, we comprehensively evaluated nine candidate reference genes i.e. *RPS17*, *RPL32*, *eEF1-γ*, *eIF2α*, *PGK1*, *PP2A*, *ILK*, *ACT* and *STK* at fourteen points of development as well as C6/36 cell culture. Adults are further divided by sex and blood meal status. The three algorithms of BestKeeper, geNorm, and NormFinder were used to analyse their stability and to rank the candidate genes in order of favourability for usage within any biological sample. The suitable reference gene(s) suggested through the outcomes of this study can be applied for normalization of qPCR data for whole organism *Ae*. *albopictus* tissue at multiple developmental stages as well as cell culture.

## Materials and methods

### Rearing and sample collection

Dried viable eggs of VCRU-lab strain *Ae*. *albopictus* were obtained from Vector Control Research Unit, Science University of Malaysia (USM). Roughly 500 were submerged in dechlorinated water at any one time. First instar larvae (1L) samples were collected immediately upon hatching. Hatchlings were maintained in relative humidity and natural light conditions at 28°C in plastic containers throughout the entire span of their aquatic life-cycle. Rearing water is changed every other day. Feed of approximately 1.0g mixture of crushed dog biscuits and pulverized chicken liver was administered daily. Second (2L), third (3L), and fourth (4L) instar individuals were collected successively. Pupal samples were a mixture of an equal number of individuals at first, second, and third day of pupation. Newly-eclosed adults were transferred to cages. They were maintained on 10% sucrose solution. Combined adult samples collected comprised of equal numbers of males and non-blood fed females aged 1 to 10 DAE. Adults of the same age range were also gender-separated in sampling. Food was removed from 5 to 7 DAE adults a full day before blood-feeding on lab-strain mice. Mosquitoes were returned to normal breeding conditions for females to lay eggs. Embryos were collected at three-hour intervals up to 12 hours; six-hour intervals from 12 to 24 hours; and 24-hour intervals from 24 to 72 hours after they were laid. C6/36 cells cultured in Gibco^®^ L-15 media supplemented with 10% FBS, 1% Pen-Strep and 10% Tryptose Phosphate Broth (ThermoFisher Scientific, USA) were harvested at maximum confluency.

### RNA extraction and quality assurance

This study adheres to the Minimum Information for Publication of Quantitative Real-Time PCR guidelines or MIQE. The amount of tissue collected per bioreplicate are as follows: ~500 eggs per embryonic sample; 50 individuals per first or second instar larval sample; 35 individuals per third larval sample; 20 individuals per fourth instar larvae or pupal sample; 20 individuals per adult sample; and 2ml of 4^th^ day culture per cell sample. All samples were immediately stored -20°C in TRIzol^®^ reagent (Invitrogen^™^, Ambion^™^, Life Technologies). Total RNA extraction was done within five days of collection with a protocol previously described for mosquito tissue samples. Culturing media was removed from c6/36 samples prior to RNA extraction as described by Abcam^®^. Extracts were quantified on the Quawell^®^ Q3000 UV Spectrophotometer (Quawell Technology, Inc., California). The acceptable A260:A280 value was set between 1.75 and 2.05. All extracts used showed clear 18S banding and minimal smearing in 1.0% agarose gel, and were kept at -20°C for the duration of the experiment.

### Reference gene selection, primer design, and primer validation

Genes were chosen if they fulfil either one of the following criteria: (a) potential as a good reference gene based on entomological literature (b) availability of an annotated sequence, or (c) at least 95% identical homology with annotated *A*. *aegypti* NCBI RefSeq. All primer pairs were designed on the Primer3 software (bioinfo.ut.ee/primer3-0.4.0/). The Kalign nucleic acid alignment software (http://www.ebi.ac.uk/Tools/msa/kalign/) was used to specify for regions spanning exon-exon boundaries. Restrictive parameters for primer selection were: melting temperatures between 59.0°C and 61.0°C, GC content between 40 and 60%, nucleotide length between 18 and 24, and amplicon length of between 150 to 225 bases. Other settings were kept at default. Singular amplicon product and mRNA specificity was confirmed *in silico* on the Sequence Manipulation Suite website (http://www.bioinformatics.org/sms2/). All genes, accession numbers, primer sequences and amplicon size used for this study is listed in Table 1.

**Table 1:** Specifications and amplification characteristics of candidate genes.

Gene	NCBI accession no.	Primer sequence	Amplicon size (bp)	Ct range	Std. Error	R2	E%
*RPS17 (40S Ribosomal Protein S17)*	AALF016139-RA	FW 5’ GAACGACAGCAGCGAAACTT	192	14.25–22.80	1.944	0.998	97.2
		RV 5’ GTCACGAAACCAGCGATCTT					
*PP2A (Protein Phosphatase 2A)*	AALF009837-RA	FW 5’ TGTGTACGACCTGTTCTTGAGG	160	20.67–29.15	1.804	0.995	105
		RV 5’ CACCCGATGAAGGACAGTCT					
*eEF1-γ (Eukaryotic Elongation Factor 1-Gamma)*	AALF027751-RA	FW 5’ GGAAAGGTCCCCGCATT	159	23.84–29.89	1.412	0.977	117.1
		RV 5’ CGGCAGCAGTTCATTGTC					
*RPL32 (60S Ribosomal Protein L32)*	AALF014686-RA	FW 5’ TATGACAAGCTTGCCCCCAA	146	14.97–24.34	2.083	0.994	96.9
		RV 5’ AGGAACTTCTTGAATCCGTTGG					
*PGK1 (Phosphoglycerate Kinase 1)*	AALF007981-RA	FW 5’ TGGAAAATGTCCGATTCTACG	179	18.75–29.67	2.268	0.972	103.1
		RV 5’ GCCCATCATTGAACTGTGC					
*ILK (Integrin-Linked Kinase)*	AALF017749-RA	FW 5’ CTTTAGTCCATTGCACTGGTG	189	23.61–33. 86	2.192	0.991	103.2
		RV 5’ TTGGCAGCGTTCACATCC					
*STK (Serine-Threonine Kinase)*	AALF009209-RA	FW 5’ TGCTATTAAGGTGATGCGCAAATC	166	21.84–29. 20	1.647	0.993	95
		RV 5’ CACCATGTACTCCATCACCAG					
*eIF2a (Eukaryotic Initiation Factor 2-Alpha)*	AALF024907-RA	FW 5’ TGAAGTTCACCAACGAGCAG	196	21.75–30.66	2.034	0.982	104.8
		RV 5’ GTTGCTCAGCAGCAGTTCCT					
*ACT* (*Actin*)	Tortosa et al., 2008	FW 5’ GCAAACGTGGTATCCTGAC	135	18.98–28.70	2.940	0.99	98.9
		RV 5’ GTCAGGAGAACTGGGTGCT					

### Reverse transcription and qPCR

Reverse transcription was carried out with 1.5μg total RNA in 30μl reactions using the iScript Reverse Transcription Supermix (Bio-Rad Laboratories, California; cat. no. 1708840) according to manufacturer’s protocol. Pooled undiluted cDNA from all eleven developmental stages were serially diluted to the factor of 5 (1:1, 1:5, 1:25, 1: 125, 1:625) for standard curve generation. All qPCR runs were performed on the BioRad CFX96 qPCR platform. Optimum qPCR reactions were in 10μl reactions of iTaq^™^ Universal SYBR^®^ Green Supermix (Bio-Rad Laboratories, California; cat. no. 1725120), ~10ng total cDNA, and 500nM each of forward and reverse primers. The standard protocol is initial denaturation at 95°C for 2.30mins, followed by 40 cycles of denaturation at 95°C for 15s, annealing at 59°C for 15s and extension at 72°C for 20s. A melting-curve analysis with a temperature range of between 65°C and 95°C immediately followed amplification. All samples were quantified in technical triplicates. Expression levels were recorded as cycle threshold (Ct). Efficiency values (E) were calculated according to the equation: E = (10^[-1/slope]^-1) X 100.

### Data mining and selection of reference gene candidates with algorithms: geNorm, BestKeeper, and NormFinder

Publicly available evaluation tools i.e. BestKeeper [35,36], geNorm [17] and Normfinder [37] were utilized for selection of best candidate gene. The BestKeeper algorithm is an Excel program which generates a ranking through repeated pairwise correlation and regression analysis of a gene against all the other tested candidates. Raw data of Ct values (annotated as CP) and PCR efficiency of the primers were used to determine the correlation between each candidate gene and the index, expressed in the form of a coefficient of determination. For geNorm and NormFinder, raw data was converted into linear values relative to the lowest Ct recorded for each candidate gene. In geNorm, the stability of a gene is assessed through the consistency of its expression ratio across all samples. The software generates both a stability value i.e. M, and a pairwise variation value i.e. V. M represents the average variation in transcript levels of a gene in comparison to all other candidate genes, achieved through a repeated process of stepwise exclusion commencing from the least stable gene. Pairwise variation estimates the effect of including another gene [17] sequentially as per the established M-value rankings through the formula of V_n_/V_n_+1. A threshold of 0.15 is set; a V value below this would mean that an additional reference gene would not improve normalization. NormFinder is a mixed-effects model statistical analysis which estimates the stability value of a gene as a function of the approximate expression variation it would impose onto the target gene data during normalization [37]. The lower this value is, the less variation one would introduce to a normalization exercise should the candidate gene be used as a reference. It also estimates the variation between sample subgroups of the sample set. The *BestKeeper* vs. *Pearson* correlation coefficient value, geNorm M value, and NormFinder stability value are perceived as weightage. Geometric means i.e. central tendencies of these weightages for a candidate gene forms the basis for generation of a consensus ranking.

## Results

### Primer pair evaluation of candidate reference genes

The expression patterns of an upwards of sixteen housekeeping genes were initially observed from previously reported RNAseq data [38,39]. Seven genes including *Ribosomal Protein L34* (*RPL34*), *α-tubulin*, *β-tubulin*, *RNA Polymerase II* (*RNAPII*), *18S*, *TATA-Box Binding Protein* (*TBP*), and *Ribosomal Protein S7* (*RPS7*) were eventually excluded due to any one or more of the following factors: (a) lack of introns or exon-exon boundaries enabling mRNA-specificity; (b) unsuitably low or high Ct values, which compromises sensitivity; and (c) poor primer design (see S1 File). Nine shortlisted candidates progressed through to the next stages of analysis. They can be divided into four functional classes: (i) ribosomal and protein-production genes: *RPS17*, *RPL32*, *eEF1-γ*, and *eIF2α*; (ii) metabolism-related gene: *PP2A*; (iii) signal-transduction genes: *PGK1*, *ILK* and *STK*; and (iv) structural integrity gene: *ACT*. A standard curve for each primer pair was generated with pooled cDNA serially diluted by a factor of 5. Primer quality is based on the efficiency (E) and linear regression coefficient (R^2^) values as observed from the amplification of cDNA at very high to very low concentrations. All recorded acceptable E values between 95.0 and 117.1%. R^2^ values range from 0.979 to 0.998 (Table 1). Amplification specificity was displayed through the production of a singular peak in melt-curve analysis, and confirmed on a 2% agarose gel (S1 Fig). PCR products were also sequenced and confirmed to have an alignment of at least 95% to the predicted gene region. All sequences are obtainable from GenBank’s BankIt depository with the accession numbers KY199533 to KY199541.

### Expression levels and sample integrity

**Fig 1:**
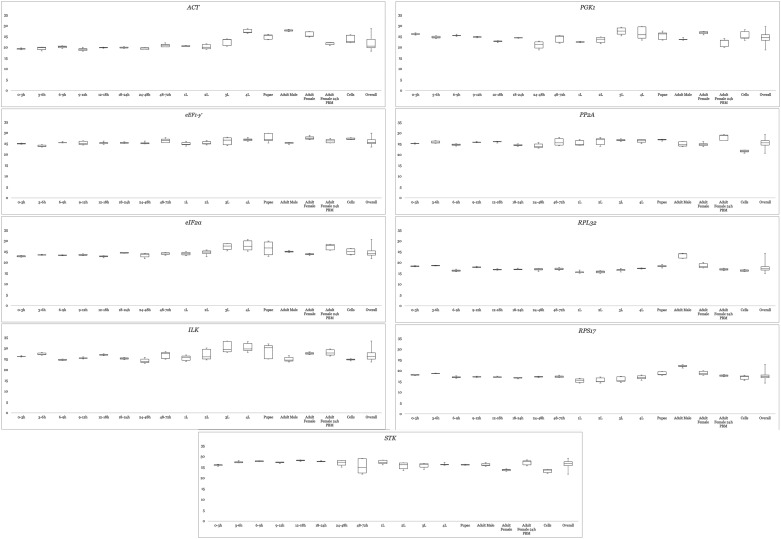
Box and whisker plot of candidate genes. Shown are the genes’ performance in individual developmental stages as well as overall performance. The ends of the box represents upper and lower quartiles; median is marked by the horizontal line inside the box.

Expression levels were quantified and candidate gene variability in any developmental stage and cell culture are displayed as Box-Whisker plots in Fig 1. *RPS17* recorded the lowest Ct value at 14.25, whereas the highest was by *ILK* with a reading of 33.86. The mean Ct values of candidates classifies the genes into two groups: (A) genes expressed at moderate levels, and (C) genes expressed at moderately-low levels. Group A genes *RPS17* and *RPL32* both recorded Ct means of 17.49. The remaining genes belong to Group B. Their mean Ct values are 24.52, 24.67, 25.46, 25.83, 26.39, and 26.74 for *PGK1*, *eIF2α*, *PP2A*, *eEF1-γ*, *STK*, and *ILK*, respectively. Sample integrity is inferred from the intrinsic variation (InVar) score as generated by the BestKeeper algorithm. Removal of samples with scores in excess of ±3.0 is recommended [36]. Low InVar scores seen across the board signifies that triplicate variability was acceptable amongst the samples of each developmental stage.

### BestKeeper analysis

BestKeeper estimates the standard deviation (SD) value of each candidate gene from raw Ct numbers. An SD>1 signifies that the variations in expression of a gene within a sample of the same origin are high, and thus indicating its instability. Our data demonstrated that not all candidates were stable across all samples (see S1 Table). *ILK* and *PGK1* frequently appeared to be unreliable. Both gave an SD above the acceptable threshold in 48 to 72 hour embryos, larval samples from second to the fourth instar stages, pupal samples, and blood-fed females. *PGK1* were additionally unstable in 24 to 48 hour embryos and cell culture. *STK* displayed instability in embryos 24 to 72 hours in age as well as second instar larvae; *PP2A* in 48 to 72 hour embryos, second instar larvae, and blood-fed females; *eEF1-γ* in in third instar larvae and pupae; *ACT* in third instar larvae and cell culture; and *eIF2α* in third and fourth instar larval samples, pupae, and blood-fed females. Both ribosomal-linked genes i.e. *RPS17* and *RPL32* were stable in all developmental time points. The SD of a gene factors into its position on a ranking based on the value given as *BestKeeper* vs *Pearson* correlation of coefficient. The closer this value is to 1, the greater the reliability of the gene. Here, those carrying SD values of above 1 are relegated to the bottom of the table regardless of its correlation of coefficient value. BestKeeper most recommends *ACT* for 24 to 48 hour embryos, second instar larvae and blood-fed adult females; *eEF1-γ* for 18 to 24 hour embryos; *eIF2α* for each of the 18 to 24 hour and 48 to 72 hour embryonic stages along with cell culture samples; *ILK* for 0 to 3 hour, 3 to 6 hour, and 9 to 12 hour embryos; *PGK1* for 6 to 9 hour embryos; *PP2A* from the fourth instar larval stage through male adulthood; *RPL32* in third instar larvae; and finally *RPS17* during the first instar larval period as well as non-blood fed females. Rankings are shown in Table 2.

**Table 2:** Ranking of candidate genes based from BestKeeper.

Rank	Developmental Stage								
	**0-3h**	**3-6h**	**6-9h**	**9-12h**	**12-18h**	**18-24h**	**24-48h**	**48-72h**	**1L**
1	*ILK*	*ILK*	*PGK1*	*ILK*	*eIF2α*	*eEF1-γ*	*ACT*	*eIF2α*	*RPS17*
2	*ACT*	*PGK1*	*RPS17*	*STK*	*PP2A*	*ACT*	*RPS17*	*RPL32*	*eEF1-γ*
3	*PP2A*	*STK*	*PP2A*	*ACT*	*PGK1*	*PGK1*	*ILK*	*RPS17*	*ILK*
4	*PGK1*	*PP2A*	*RPL32*	*eEF1-γ*	*ILK*	*ILK*	*PP2A*	*eEF1-γ*	*STK*
5	*RPS17*	*eEF1-γ*	*STK*	*PGK1*	*eEF1-γ*	*STK*	*RPL32*	*ACT*	*RPL32*
6	*eIF2α*	*eIF2α*	*ILK*	*eIF2α*	*STK*	*eIF2α*	*eEF1-γ*	*PGK1*	*ACT*
7	*eEF1-γ*	*ACT*	*ACT*	*PP2A*	*ACT*	*RPS17*	*eIF2α*	*ILK*	*PGK1*
8	*RPL32*	*RPL32*	*eEF1-γ*	*RPL32*	*RPL32*	*PP2A*	*STK*	*STK*	*eIF2α*
9	*STK*	*RPS17*	*eIF2α*	*RPS17*	*RPS17*	*RPL32*	*PGK1*	*PP2A*	*PP2A*
	**2L**	**3L**	**4L**	**Pupae**	**Adult, Male**	**Adult, Female**	**Adult, Female, 24h PBM**	**C6/36 cells**	
1	*ACT*	*RPL32*	*PP2A*	*PP2A*	*PP2A*	*RPS17*	*ACT*	*eIF2α*	
2	*RPL32*	*STK*	*eEF1-γ*	*RPL32*	*STK*	*eEF1-γ*	*eEF1-γ*	*STK*	
3	*eEF1-γ*	*RPS17*	*RPL32*	*STK*	*ILK*	*RPL32*	*STK*	*eEF1-γ*	
4	*RPS17*	*PP2A*	*RPS17*	*ACT*	*eEF1-γ*	*ACT*	*RPL32*	*RPL32*	
5	*eIF2α*	*eEF1-γ*	*ACT*	*RPS17*	*PGK1*	*PGK1*	*RPS17*	*RPS17*	
6	*ILK*	*ACT*	*STK*	*ILK*	*RPS17*	*STK*	*eIF2α*	*PP2A*	
7	*PGK1*	*ILK*	*eIF2α*	*eIF2α*	*RPL32*	*PP2A*	*PGK1*	*ILK*	
8	*STK*	*PGK1*	*PGK1*	*PGK1*	*ACT*	*eIF2α*	*PP2A*	*PGK1*	
9	*PP2A*	*eIF2α*	*ILK*	*eEF1-γ*	*eIF2α*	*ILK*	*ILK*	*ACT*	

Genes highlighted red had an SD of over the recommended stability indicator of 1. BestKeeper values (not shown here; please refer S1 Table for more information) are based on the r-value, i.e. the *BestKeeper* vs. *Pearson* coefficient of correlation.

### geNorm analysis

**Fig 2:**
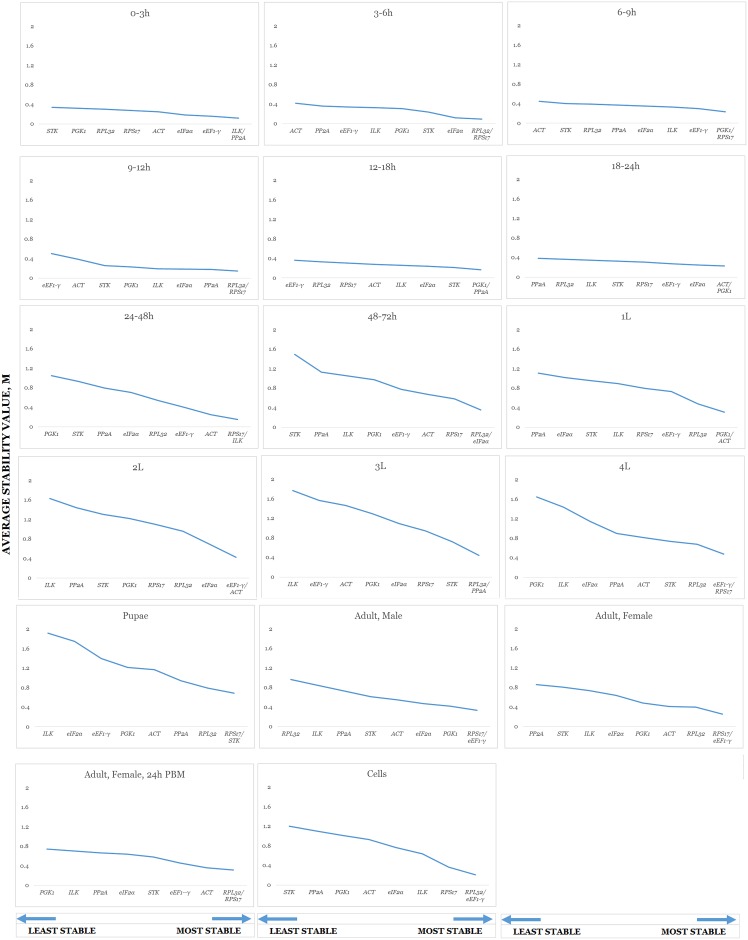
Average stability values (M) of genes in individual developmental stages and cell culture. Lower values indicate better stability.

**Fig 3:**
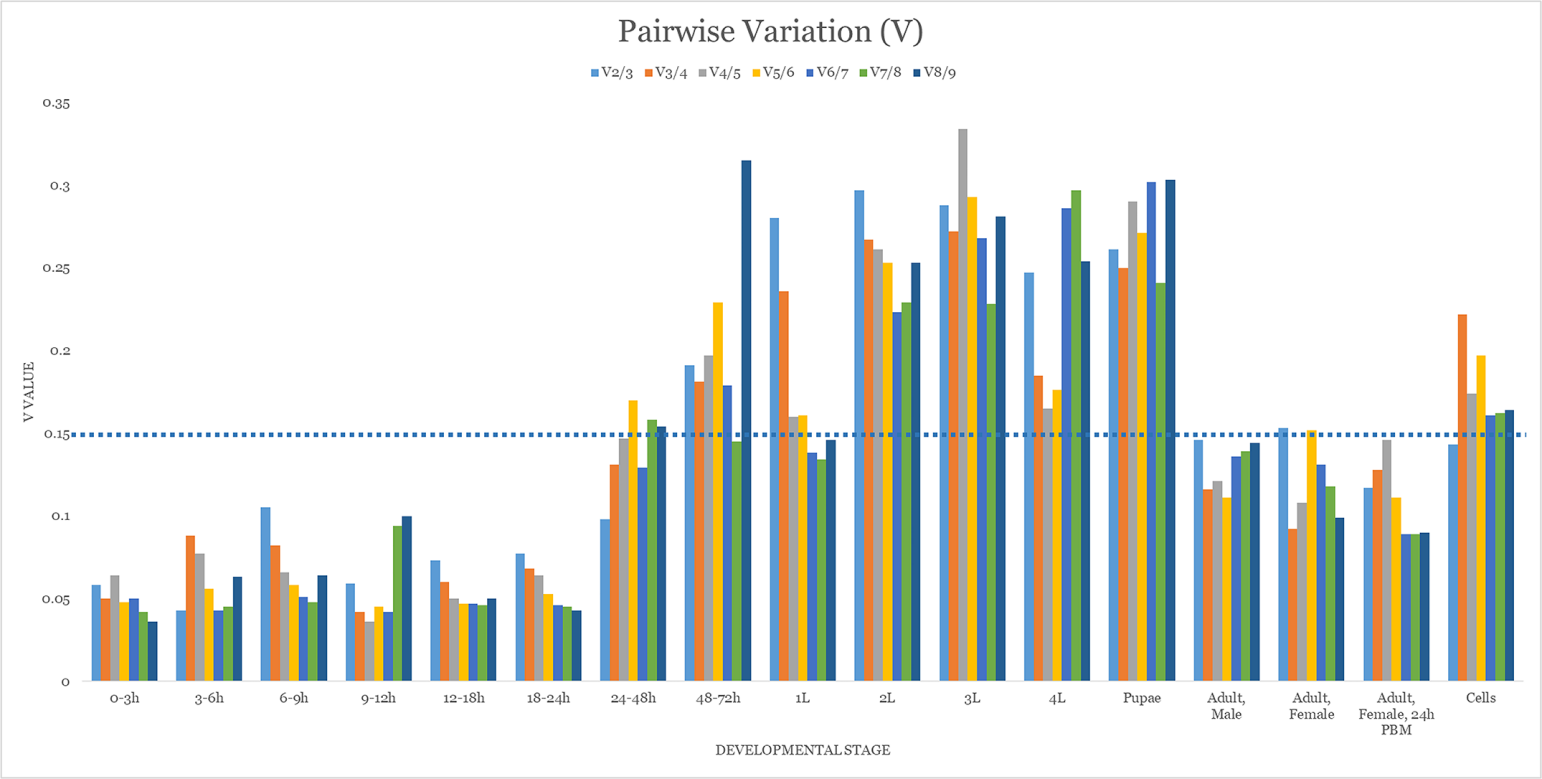
Pairwise variation (V) analysis of candidate reference genes. A pairwise variation suggest that the added gene would improve normalization and should preferably be included in normalization. The proposed cut-off value is 0.15.

geNorm provides two assessment outcomes. The first is an average expression stability score as symbolized by M. The higher the M-value of a gene, the less stable it is perceived to be. This value should fall below 1.5. None of the candidate genes exceeded this in any developmental stage (Table 3). As considerations for a pairing of genes for normalization purposes is incorporated into the algorithm, two are viewed as equally stable or applicable for any single stage. An *ILK* and *PP2A* pairing is suggested for 0 to 3 hour embryos; *RPL32* and *RPS17* for the 3 to 6 hour and 9 to 12 hour embryonic stages as well as blood-fed females; *PGK1* each with *RPS17* for 6 to 9 hour embryos, with *PP2A* for the 12^th^ to 18^th^ hour, and with *ACT* for 18 to 24 hour embryos along with the first instar larval stage. *RPS17* is to be paired with *ILK* for 24 to 48 hour embryonic samples; with *eEF1-γ* for fourth instar larvae and both sexes in non-blood fed adults; and with *STK* for pupal samples. Second instar larval samples is best normalized by an *ACT* and *eEF1-γ* pairing. For each of the remaining developmental time points i.e. 48 to 72 hour embryos, third instar larvae, and cell culture, *RPL32* provides fairest equilibration when paired with *eIF2α*, *PP2A*, and *eEF1-γ*, respectively. Rankings are summarized in charts as provided by the software (Fig 2). The second outcome from geNorm estimates the effect of a gene addition event [17]. The proposed cut-off value, denoted as V, is 0.15. Our data shows that all samples of larval and pupal tissue, as well as those of 48 to 72 hour embryos and non-blood fed female adults, should require more than two candidate genes to be properly normalized (Fig 3).

**Table 3:** Rankings of candidate genes by geNorm.

Rank	Developmental stage											
	**0-3h**		**3-6h**		**6-9h**		**9-12h**		**12-18h**		**18-24h**	
1/2	*ILK/PP2A*	0.118	*RPL32/RPS17*	0.093	*PGK1/RPS17*	0.233	*RPL32/RPS17*	0.146	*PGK1/PP2A*	0.168	*ACT/PGK1*	0.228
3	*eEF1-γ*	0.160	*eIF2α*	0.123	*eEF1-γ*	0.301	*PP2A*	0.177	*STK*	0.212	*eIF2α*	0.246
4	*eIF2α*	0.189	*STK*	0.240	*ILK*	0.334	*eIF2α*	0.185	*eIF2α*	0.239	*eEF1-γ*	0.276
5	*ACT*	0.250	*PGK1*	0.307	*eIF2α*	0.352	*ILK*	0.195	*ILK*	0.257	*RPS17*	0.310
6	*RPS17*	0.275	*ILK*	0.330	*PP2A*	0.371	*PGK1*	0.228	*ACT*	0.280	*STK*	0.328
7	*RPL32*	0.305	*eEF1-γ*	0.342	*RPL32*	0.387	*STK*	0.256	*RPS17*	0.305	*ILK*	0.343
8	*PGK1*	0.323	*PP2A*	0.362	*STK*	0.403	*ACT*	0.384	*RPL32*	0.329	*RPL32*	0.364
9	*STK*	0.339	*ACT*	0.418	*ACT*	0.450	*eEF1-γ*	0.505	*eEF1-γ*	0.364	*PP2A*	0.382
	**24-48h**		**48-72h**		**1L**		**2L**		**3L**		**4L**	
1/2	*RPS17/ILK*	0.153	*RPL32/eIF2α*	0.354	*PGK1/ACT*	0.310	*eEF1-γ/ACT*	0.422	*RPL32/PP2A*	0.444	*eEF1-γ/RPS17*	0.477
3	*ACT*	0.251	*RPS17*	0.585	*RPL32*	0.476	*eIF2α*	0.692	*STK*	0.723	*RPL32*	0.678
4	*eEF1-γ*	0.398	*ACT*	0.670	*eEF1-γ*	0.729	*RPL32*	0.955	*RPS17*	0.944	*STK*	0.738
5	*RPL32*	0.541	*eEF1-γ*	0.778	*RPS17*	0.797	*RPS17*	1.094	*eIF2α*	1.091	*ACT*	0.815
6	*eIF2α*	0.709	*PGK1*	0.970	*ILK*	0.896	*PGK1*	1.220	*PGK1*	1.294	*PP2A*	0.900
7	*PP2A*	0.799	*ILK*	1.051	*STK*	0.955	*STK*	1.305	*ACT*	1.466	*eIF2α*	1.146
8	*STK*	0.935	*PP2A*	1.128	*eIF2α*	1.016	*PP2A*	1.437	*eEF1-γ*	1.565	*ILK*	1.436
9	*PGK1*	1.050	*STK*	1.495	*PP2A*	1.106	*ILK*	1.631	*ILK*	1.766	*PGK1*	1.646
	**Pupae**		**Adult, Male**		**Adult, Female**		**Adult, Female, 24h PBM**		**Cells**			
1/2	*RPS17/STK*	0.689	*RPS17/eEF1-γ*	0.334	*RPS17/eEF1-γ*	0.253	*RPL32/RPS17*	0.314	*RPL32/eEF1-γ*	0.211		
3	*RPL32*	0.791	*PGK1*	0.419	*RPL32*	0.400	*ACT*	0.360	*RPS17*	0.365		
4	*PP2A*	0.941	*eIF2α*	0.469	*ACT*	0.413	*eEF1-γ*	0.455	*ILK*	0.637		
5	*ACT*	1.167	*ACT*	0.550	*PGK1*	0.486	*STK*	0.584	*eIF2α*	0.765		
6	*PGK1*	1.216	*STK*	0.614	*eIF2α*	0.637	*eIF2α*	0.638	*ACT*	0.929		
7	*eEF1-γ*	1.396	*PP2A*	0.729	*ILK*	0.739	*PP2A*	0.666	*PGK1*	1.013		
8	*eIF2α*	1.748	*ILK*	0.850	*STK*	0.810	*ILK*	0.704	*PP2A*	1.104		
9	*ILK*	1.917	*RPL32*	0.963	*PP2A*	0.859	*PGK1*	0.746	*STK*	1.200		

Scores displayed are M values. The two top genes share the same value. A lower value denotes greater stability. M should not exceed 1.5.

### NormFinder analysis

Similar to geNorm, the data utilized by this program is based on relative values. The algorithm produces a stability value for each gene when compared to others within the group. A lower value indicates greater stability. The program does not make suggestions for a cut-off value [37]. Rankings generated are summarized in Table 4. *eEF1-γ* is deemed the best reference gene for the 6 to 9 hour embryonic stage and both male and blood-fed female adults; *PGK1* for non-blood fed females, 9 to 12 hour and 12 to 18 hour embryos, and first instar larval samples; *PP2A* for 0 to 3 hour and 12 to 18 hour embryonic tissue as well as pupal samples; *RPL32* for 48 to 72 hour embryos, cell culture, and larval samples of the second through fourth instar; *RPS17* for 24 to 48 hour embryos, and finally *STK* for those aged between 3 to 6 hours. Despite the frequency at which *PGK1* is top-ranked, it is also the most commonly disrecommended candidate (n = 4/17). In the absence of group identifiers, it is presumed that the two genes with the lowest stability value within a sample set would provide the best combination for two-reference gene normalization strategies [40].

**Table 4:** NormFinder analysis and established rankings.

Rank	Developmental stages											
	**0-3h**		**3-6h**		**6-9h**		**9-12h**		**12-18h**		**18-24h**	
1	*PP2A*	0.097	*STK*	0.125	*eEF1-ƴ*	0.132	*PGK1*	0.111	*PP2A*	0.069	*PGK1*	0.079
2	*RPS17*	0.119	*PGK1*	0.150	*eIF2α*	0.143	*STK*	0.117	*eIF2α*	0.129	*eIF2α*	0.112
3	*ILK*	0.130	*eIF2α*	0.153	*ILK*	0.154	*ILK*	0.149	*ACT*	0.138	*ACT*	0.143
4	*ACT*	0.152	*ILK*	0.154	*PGK1*	0.192	*RPS17*	0.153	*STK*	0.142	*eEF1-ƴ*	0.184
5	*eEF1-ƴ*	0.159	*RPL32*	0.195	*STK*	0.209	*PP2A*	0.160	*PGK1*	0.152	*RPS17*	0.199
6	*RPL32*	0.198	*eEF1-ƴ*	0.200	*RPS17*	0.225	*eIF2α*	0.217	*ILK*	0.170	*STK*	0.203
7	*eIF2α*	0.208	*RPS17*	0.237	*PP2A*	0.244	*RPL32*	0.233	*RPS17*	0.201	*ILK*	0.236
8	*STK*	0.219	*PP2A*	0.250	*RPL32*	0.278	*ACT*	0.437	*RPL32*	0.275	*RPL32*	0.251
9	*PGK1*	0.233	*ACT*	0.386	*ACT*	0.391	*eEF1-ƴ*	0.618	*eEF1-ƴ*	0.306	*PP2A*	0.259
	**24-48h**		**48-72h**		**1L**		**2L**		**3L**		**4L**	
1	*RPS17*	0.053	*RPL32*	0.133	*PGK1*	0.107	*RPL32*	0.199	*RPL32*	0.075	*RPL32*	0.152
2	*ACT*	0.089	*eIF2α*	0.133	*ACT*	0.107	*eEF1-γ*	0.574	*PP2A*	0.154	*PP2A*	0.267
3	*ILK*	0.124	*RPS17*	0.155	*RPL32*	0.374	*PGK1*	0.741	*STK*	0.877	*STK*	0.272
4	*eEF1-γ*	0.440	*ACT*	0.337	*eEF1-γ*	0.535	*eIF2α*	0.783	*RPS17*	0.900	*ACT*	0.636
5	*RPL32*	0.545	*eEF1-γ*	0.641	*RPS17*	0.561	*ACT*	0.927	*PGK1*	0.923	*eEF1-γ*	0.805
6	*PP2A*	0.621	*PGK1*	0.900	*eIF2α*	0.599	*RPS17*	0.963	*eIF2α*	0.963	*RPS17*	0.839
7	*STK*	0.718	*ILK*	0.972	*STK*	0.712	*PP2A*	1.097	*ACT*	1.252	*ILK*	1.437
8	*eIF2α*	0.806	*PP2A*	1.068	*ILK*	0.780	*STK*	1.136	*eEF1-γ*	1.390	*eIF2α*	1.437
9	*PGK1*	0.942	*STK*	2.577	*PP2A*	0.891	*ILK*	1.547	*ILK*	1.721	*PGK1*	1.558
	**Pupae**		**Adult, Male**		**Adult, Female**		**Adult, F, 24h PBM**		**Cells**			
1	*PP2A*	0.168	*eEF1-γ*	0.170	*PGK1*	0.168	*eEF1-ƴ*	0.199	*RPL32*	0.073		
2	*RPL32*	0.240	*PGK1*	0.210	*eEF1-γ*	0.260	*eIF2α*	0.238	*eEF1-γ*	0.073		
3	*RPS17*	0.517	*eIF2α*	0.254	*RPS17*	0.281	*ACT*	0.286	*ILK*	0.377		
4	*STK*	0.580	*ACT*	0.279	*eIF2α*	0.441	*STK*	0.336	*RPS17*	0.452		
5	*ACT*	1.132	*STK*	0.334	*ILK*	0.457	*PP2A*	0.355	*eIF2α*	0.500		
6	*eEF1-γ*	1.302	*RPS17*	0.379	*RPL32*	0.517	*RPL32*	0.436	*PP2A*	0.737		
7	*PGK1*	1.491	*PP2A*	0.623	*ACT*	0.560	*ILK*	0.439	*ACT*	0.870		
8	*eIF2α*	1.667	*ILK*	0.846	*STK*	0.569	*RPS17*	0.494	*PGK1*	0.959		
9	*ILK*	1.857	*RPL32*	0.882	*PP2A*	0.599	*PGK1*	0.545	*STK*	0.999		

Numbers depict stability; the lower the value, the greater the stability.

### Consensus list of reference genes

Consensus rankings are obtained through geometrically averaging the weights assigned to a candidate gene (in the form of stability values from geNorm and NormFinder, and a function of 1-((*BestKeeper* vs. *Pearson* correlation coefficient value) from BestKeeper) by the three programs. All genes are included regardless of BestKeeper SD values. Results are summarized in Table 5. The three top-ranked genes in the consensus list for any developmental stage are typically considered to be most reliable [37]. However, as these combinations vary greatly from one developmental stage to the next, reliability across sample types is also assessed on the basis of overall frequency at which a gene appears amongst the top-three. *eEF1-γ* and *RPL32* are the most reliable, with a frequency of 0.157 and 0.137, respectively.

**Table 5:** Rankings from all three algorithms and resulting consensus.

	Developmental stage					
**Rank**	**0-3h**	**3-6h**	**6-9h**	**9-12h**	**12-18h**	**18-24h**
1	*ILK*	*PGK1*	*PGK1*	*ILK*	*PP2A*	*PGK1*
2	*PP2A*	*ILK*	*RPS17*	*STK*	*eIF2α*	*eEF1-ƴ*
3	*ACT*	*eIF2α*	*eEF1-ƴ*	*PGK1*	*PGK1*	*ACT*
4	*RPS17*	*STK*	*PP2A*	*RPS17*	*ILK*	*eIF2α*
5	*eEF1-ƴ*	*RPL32*	*ILK*	*PP2A*	*STK*	*ILK*
6	*eIF2α*	*eEF1-ƴ*	*eIF2α*	*eIF2α*	*ACT*	*STK*
7	*PGK1*	*PP2A*	*STK*	*RPL32*	*eEF1-ƴ*	*RPS17*
8	*RPL32*	*RPS17*	*RPL32*	*ACT*	*RPS17*	*PP2A*
9	*STK*	*ACT*	*ACT*	*eEF1-ƴ*	*RPL32*	*RPL32*
	**24-48h**	**48-72h**	**1L**	**2L**	**3L**	**4L**
1	*ACT*	*eIF2α*	*ACT*	*RPL32*	*RPL32*	*RPL32*
2	*ILK*	*RPL32*	*PGK1*	*ACT*	*PP2A*	*PP2A*
3	*RPS17*	*RPS17*	*RPS17*	*eEF1-γ*	*STK*	*eEF1-γ*
4	*eEF1-γ*	*ACT*	*eEF1-γ*	*eIF2α*	*RPS17*	*STK*
5	*RPL32*	*eEF1-γ*	*RPL32*	*ILK*	*eIF2α*	*RPS17*
6	*PP2A*	*PGK1*	*ILK*	*PGK1*	*PGK1*	*ACT*
7	*eIF2α*	*ILK*	*STK*	*PP2A*	*ACT*	*eIF2α*
8	*STK*	*PP2A*	*eIF2α*	*ILK*	*eEF1-γ*	*PGK1*
9	*PGK1*	*STK*	*PP2A*	*STK*	*ILK*	*ILK*
	**Pupae**	**Adult, Male**	**Adult, Female**	**Female, 24h PBM**	**C6/36 cells**	
1	*PP2A*	*eEF1-γ*	*RPS17*	*eIF2α*	*eEF1-γ*	
2	*RPL32*	*STK*	*eEF1-γ*	*ACT*	*RPL32*	
3	*STK*	*PP2A*	*RPL32*	*eEF1-ƴ*	*eIF2α*	
4	*RPS17*	*PGK1*	*PGK1*	*PGK1*	*RPS17*	
5	*ACT*	*RPS17*	*ACT*	*PP2A*	*ILK*	
6	*ILK*	*eIF2α*	*eIF2α*	*STK*	*PP2A*	
7	*eIF2α*	*ACT*	*STK*	*RPL32*	*ACT*	
8	*PGK1*	*ILK*	*ILK*	*RPS17*	*PGK1*	
9	*eEF1-γ*	*RPL32*	*PP2A*	*ILK*	*STK*	

Consensus rankings are based on the geometric means of weightages in the form of stability values from geNorm and NormFinder, and a function of 1-(*BestKeeper* vs. *Pearson* correlation coefficient value) from BestKeeper.

## Discussion

The mosquito is intensively studied for a number of reasons: the central role of several of its species as propagators of disease-causing organisms notwithstanding, the insect’s wide-travelled nature and historically paralleled evolution alongside humans have also provided insights into adaptive organogenesis and development [41,42]. *Ae*. *albopictus* is especially interesting to study as it is both a potent carrier of arboviruses and the most invasive species of mosquito to have emerged in recent years [4,43]. As recognized pests, much of the research surrounding members of the Aedes genus is concerned with their control. Unlike *A*. *aegypti* however, *Ae*. *albopictus* remains largely susceptible to commonly used pesticides [44–47]. Regardless, comparative assays and subsequent investigations into how a single pesticide could affect these two genetically similar organisms vastly differently could be the key to understanding the genetics of mechanisms of resistance, and eventually offer a solution for overcoming the growing problem of pesticide resistance in *Ae*. *aegypti*.

These studies more often than not require qPCR. The method is hugely popular as it is robust, powerful, and fast. As it is widely utilized, many considerations for obtainment of assured data is also locked in place [28]. When these guidelines are followed, qPCR data is often viewed as highly reliable. Such a consideration is the normalization of expression levels through the application of reference genes [48,49]. These are typically chosen from a group classified as ‘reference genes’. However, current opinion has shied away from the assumption that only such genes need apply as references. In fact, it is highly likely that genes most suitable as normalizers could be as particular as to be tissue-specific. For *Ae*. *albopictus*, a panel of reference genes that would satisfy each individual developmental stage is as of yet undescribed. As aforementioned, *ACT* [25] or *RPS7* [26] are often utilized, usually singularly and with little thought for the potential instability of these genes in respect to experimental variables. These practices are unadvisable; not only is the utilization of a single gene for normalization no longer accepted [50], but a misinformed selection of reference genes are known to lead to false positives or false negatives [15]. It is thus of utmost importance that this lack of information is promptly addressed. The challenge lies in the unannotated state of the *Ae*. *albopictus* transcriptome, which reduces confidence in the identity of genes of interest within the species. Nonetheless, through high-confidence sequence alignments in respect to the thoroughly annotated *A*. *aegypti* transcriptome, a group of candidate genes were chosen for validation within the context of this study. Ultimately, we hope that the findings here could act as a guide towards more careful selection of reference genes in qPCR assays involving *Ae*. *albopictus*.

A total of fifteen pre-determined stages of development in *Ae*. *albopictus* were sampled. A subgroup of female adults at 24-hours post-blood meal, a point where RNA production is doubled [51], as well as C6/36 cells are also included. Stability of nine candidate genes within each developmental stage is separately evaluated by the BestKeeper, geNorm, and Normfinder tools. The three gave largely differing results, though a level of congruence was seen amongst top three rankings in a majority of the stages considered. The gene most frequently observed within this group was *eEF1-γ*. It appeared eight times out of a possible fifty-one. The gene outperformed *RPL32* (n = 7/51), as well as *PP2A*, *ACT* and *PGK1* (each n = 6/51). In contrast, *STK* was most frequently found in the bottom-three rankings (n = 8/51), followed closely by *ILK* and *RPL32* (each n = 7/51). Given that *RPL32* was equally prominent in top three and bottom three rankings, and as inconsistencies in how most of the candidates were ranked across the consensus board are seen, it is more advisable to categorize performance on a tissue-type basis. As we are also wary of preferences in the scientific community to limit reference genes to two, the following suggestions will be composed of the pair with the best showing within each tissue-type grouping.

For embryo-derived samples up to 24h post-oviposition, *PGK1* and *ILK* is the most recommendable normalizer pairing. Both encode for kinases. Along with *STK*, they are chosen as candidates as previous validation studies have found members of this category of genes to be reliable references [52,53]. Kinases act as signal transducers. As information relay occurs around the clock, they are in constant demand, and this translates into the constitutive expression of their genes. However, the ‘reusable’ nature of these enzymatic proteins limits their numbers within the cell at any one time. This is hypothesized to assert a degree of expressional stability and subsequently, the applicability of this group of genes in normalization. Regardless, as most reference genes are regulated, their levels were shown to be affected by biological needs. It is thus important to note that both *PGK1* and *ILK* performed relatively poorly outside of the 0 to 24 hour embryo tissue-type.

Greatest reliability amongst candidate genes in embryos aged between 24 and 72 hours shifts towards ribosomal genes. This trend carries through into the larval and pupal stages as well. *RPL32* is prominent within the top-three rankings throughout these developmental periods. We therefore tentatively suggest the pairing of this gene along with *RPS17* for all tissues along this span of time. Ribosomes are the protein-generating machines of the cell system. Its two subunits, the 40S and 60S ribosomal proteins, are built from a total of over 80 components. Whereas *RPS17* is a component of the 40S subunit, *RPL32* is one of 60S’. Both are common internal control genes and have displayed stability when utilized in cell culture [54–56], plants [57,58] and mammalian tissue [59,60]. In insects, *RPL32* [61] and *RPS17* [62] were shown to be reliable references regardless of tissue origin during bodily development. Cellular activity of the evolving mosquito speeds up after the 24^th^ hour within embryos. Our raw data shows expression of either gene to be high (Mean Ct value; *RPL32 =* 16.77, *RPS17* = 16.61). When only the larval stages are considered, these values are further reduced, indicating even greater expression levels. This is the point of development whereby growth is most robust. Consequently, demand for proteins to facilitate the process is at an all-time high. Nonetheless, the kinetics of ribosomal activity is such that their components can dissociate and re-associate as needs dictate. The balancing act between availability, demand, and need here may altogether incur a stabilizing effect upon ribosomal genes and thus, their inherent usability as controls for these tissue types. This coincidentally explains why *RPL32* and *RPS17* are reasonably stable within rapidly growing cell culture as well. As C6/36 cells are also of larval origin, we are doubly confident with suggesting these two genes for normalization in context of *Ae*. *albopictus* cell culture.

Within adult tissues, another participant of protein synthesis emerged as the best reference gene. *Eukaryotic Elongation Factor 1 Gamma* or *eEF1-γ* is a subunit of the protein eEF1, which delivers aminoacylated-tRNAs to the ribosome during translation. However, the overall contribution of this highly versatile protein within the cell is much more widespread, potentially holding roles in nuclear export, proteolysis, and apoptosis, amongst others [63]. Our evaluation here marks *eEF1-γ* as superior to *eIF2α* as a candidate reference gene. At the outset of this study, our expectations were such that these two would be comparable as like *eEF1-γ*, *eIF2α* is also a multifunctional player of protein synthesis [64]. It is unclear to us as to why the latter is the more volatile gene. Nonetheless, these two should provide proper normalization when utilized for expression within cell culture and blood-fed adult female tissues. For adult males and females, *eEF1-γ* is best paired with *STK* and *RPS17*, respectively. We had also hypothesized an association between *eEF1-γ* and *ACT*, as eEF1 is known to effect actin polymerization and subsequent cytoskeletal formation [65,66]. True enough, the two were found closely placed in the consensus rankings in eight out of the seventeen sample subsets here, though unexpectedly this occurred mostly under circumstances where neither showed reliability as a normalizer.

An additional form of result supplied by geNorm is a pairwise variation (V) evaluation (Fig 3). The addition of a suitable reference gene during target gene normalization is expected to reduce the normalization factors (NF) value. However, geNorm dictates that if the stepwise inclusion event is valued at below 0.15, subsequent addition events will not positively affect normalization outcomes. The V values for our study show that in many cases, the combination of two genes is sufficient. Interestingly, the sample subsets which appear to benefit from stepwise inclusion all belong to the ‘rapid-growth’ group i.e. 48 to 72 hour embryos through to pupae. Where the inclusion of none of the genes evaluated would satisfy the 0.15 threshold value, it was proposed that three would be the ideal number to use during qPCR [67,68]. For this reason, in addition to the pair of genes suggested for usage on a tissue-type basis above, we recommend the addition of a third gene most top-ranked for an individual subset as seen Table 5. Nonetheless, this observation also alludes to there being a panel of reference genes more suitable for this period of development than the ones evaluated here.

In *Aegypti*, we were able to recommend a two-gene combination of *RPS17* and *ACT* as a normalization strategy across all tissue-types on the basis of their inherent stability throughout development [62]. For *Albopictus*, *eEF1-γ* and *RPL32* instead are the genes most commonly seen in top-three rankings within the consensus. Despite this, *eEF1-γ* is also seen as the least reliable gene in two stages i.e. 9 to 12h embryos and pupae. Similarly, *RPL32* is as equally ‘stable’ as it is ‘unstable’. Therefore, we cannot assuredly advise on a two-gene combination which would be suitable enough for general use regardless of tissue-type and experimental conditions.

The overall middling performance of *ACT* is another surprising outcome of the analysis, given the gene’s history as a ubiquitously used control in not only qPCR, but also proteomics. Its stability within the developing *Ae*. *albopictus* was however only on par to the equally middling *PP2A*. We had included the phosphatase in this study given its verified usability as a reference gene during development in plants [69–71]. In animal tissue, it is less commonly studied for this purpose. Our results suggest that although *PP2A* could be applied effectively as a reference for six tissue types, the lack of obvious trends to its stability renders the gene unpredictable. This emphasizes the need for a re-evaluation of suitability of genes as references on a species-by-species basis. Improvements to be made in the future include broadening the classes of genes evaluated as well as putting variation-contributing factors such as viral infection or environmental stresses under parallel consideration. In vastly researched species such as *Drosophila*, a large panel of reference genes has also been shown to benefit normalization as sample size and experimental complexity grows [72–74].

## Conclusion

This is the first study of its kind in *Ae*. *albopictus*. Through the algorithms of BestKeeper, geNorm, and NormFinder, we recommend the implementation of two-gene combinations to provide satisfactory normalization for the described developmental stages and C6/36 cell culture on the basis of tissue-type. Based on consensus rankings, the proposed combinations are *PGK* and *ILK* for early embryos (0 to 24 hours post-oviposition); *RPL32* and *RPS17* for late embryos (24 to 72 hour post-oviposition) as well as all four larval instars and pupae samples; *eEF1-γ* with *STK* for adult males; *eEF1-γ* with *RPS17* for non-blood fed females; and *eEF1-γ* with *eIF2α* for both blood-fed females and cell culture. Application of an additional gene during the span of development from 48 to 72 hours post-oviposition embryos to the pupal stage comes highly recommended. These findings will benefit normalization practices in *Ae*. *albopictus*, and may additionally serve as a resource for screening reference genes in closely-related insects.

## Supporting information

S1 Fig PCR products in 2% agarose gel. In flanking lanes are 100bp ladder.
(PDF)

S1 File Expression level comparisons between candidate genes, and discarded primers. Table 1 is a summary of candidate gene expression levels and performance from previous publications; Table 2 is a list of primers eventually excluded from the study.
(DOCX)

S1 Table BestKeeper descriptive statistic analysis. A complete analysis of candidate genes performance by BestKeeper.
(DOCX)
