# Supplementary material for: Assessment of Aedes albopictus reference genes for quantitative PCR at different stages of development
Source: PLoS One. 2018 Mar 19;13(3):e0194664. doi: 10.1371/journal.pone.0194664 (PMC5858815; doi:10.1371/journal.pone.0194664)

Supplementary Figure S1: PCR products. Shown in this 2% agarose gel are the expected sizes of PCR products of each candidate gene. Flanking lanes are filled with 100bp ladder.

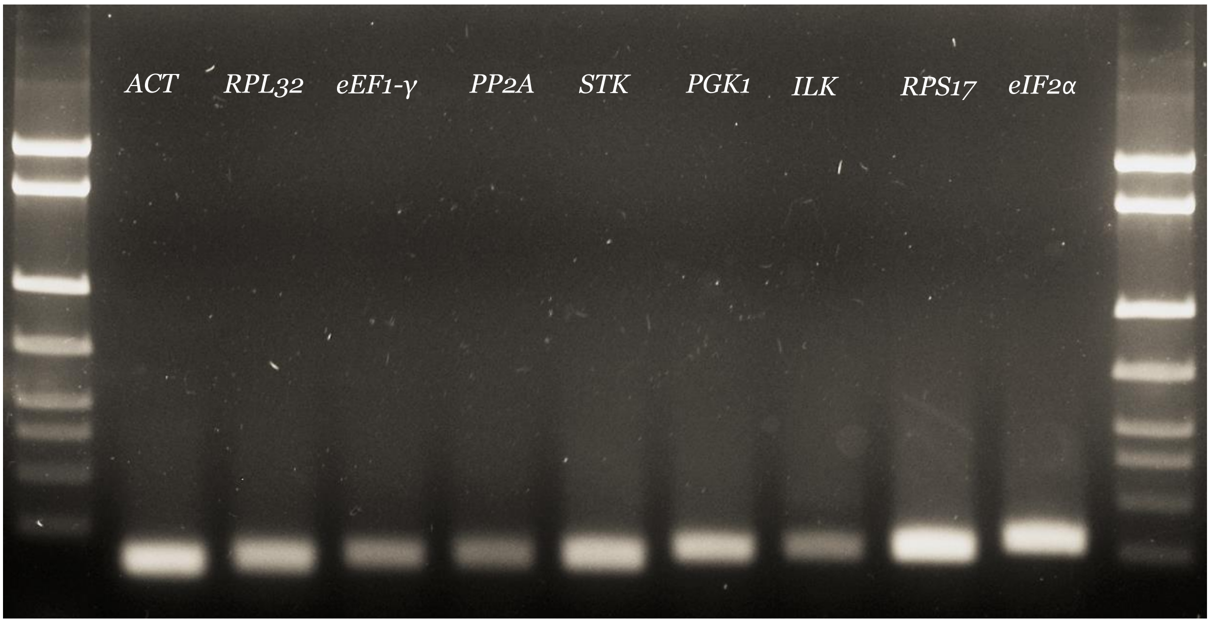

Supplement: S1 Fig — In flanking lanes are 100bp ladder. (PDF) [file pone.0194664.s001.pdf]
