# Supplementary material for: Assessment of Aedes albopictus reference genes for quantitative PCR at different stages of development
Source: PLoS One. 2018 Mar 19;13(3):e0194664. doi: 10.1371/journal.pone.0194664 (PMC5858815; doi:10.1371/journal.pone.0194664)
Supplement: S1 File — Table 1 is a summary of candidate gene expression levels and performance from previous publications; Table 2 is a list of primers eventually excluded from the study. (DOCX) [file pone.0194664.s002.docx]

| Paper  Gene | Poelchau et al. 2013([*1*](#_ENREF_1))  (growth condition and age) | Huang et al. 2015([*2*](#_ENREF_2))  (developmental stage and age) | Esquivel et al. 2016([*3*](#_ENREF_3))  (growth condition) | Tsujimoto et al. 2017([*4*](#_ENREF_4))  (age and compound) |
| --- | --- | --- | --- | --- |
| *RPS17* |  | ☑ | ☑ | ☑ |
| *RPL32* |  | ☑ | ☑ | ☑  (carcass) |
| *ACT* |  | ☑ | ☑ | ☑ |
| *ILK* | ☑ |  |  |  |
| *STK* |  | ☑ |  | ☑  (carcass) |
| *PGK1* | ☑ | ☑ | ☑ | ☑ |
| *EF1-ƴ* |  | ☑ | ☑ | ☑ |
| *IF2α* | ☑ | ☑ | ☑ | ☑  (midgut) |
| *PP2A* |  |  | ☑ |  |
| genes excluded from study | | | | |
| *H3* | ☑ | ☑ |  | ☑  (carcass) |
| *RPL10* |  | ☑ | ☑ | ☑ |
| *RPL34* |  |  |  | ☑  (carcass) |
| *α-tubulin* |  | ☑ |  |  |
| *β-tubulin* |  | ☑ | ☑ | ☑  (carcass) |

**Supplementary Information 1**

**Table 1:** The following table summarizes expression level changes of the genes assessed in this study, as well as the genes eventually excluded from the panel. Ticks indicate that the gene, based on the RNAseq data made available by the corresponding paper, was significantly differentially expressed under the conditions of the study

**Table 2:** Primer pairs designed for this study, but was eventually disregarded due to poor primer design and undesirable amplification properties

| **Gene**  **(Vectorbase ID)** | **Primer pair** | **R^2^**  **^(geomean of three bioreplicate runs)^** | **E%**  **^(geomean of three bioreplicate runs)^** |
| --- | --- | --- | --- |
| ***RPL34***  (AALF003533) | Fw 5’ AGAAGCTCAGCGGAATCAAG  Rv 5’ CTTCAGCACCTTGACGACCT | 0.995 | 84.5% |
| ***18S***  (X57172.1) | Fw 5’ ACCACATCCAAGGAAGGCAG  Rv 5’ ACTTGCCCTCCACTTGATCC | 0.776 | 119.2% |
| ***RNAPII subunit***  (AALF024200) | Fw 5’ GGACCCGGATTCGTTGGTAT  Rv 5’ ACGATGTGGGTCAGTTCGTC | 0.998 | 198.2% |
| ***α-tubulin***  (AALF007532)  1^st^ try | Fw 5’CGCATTCAGTCCTCAGAACA  Rv 5’ACACCAATCGACGAACATGA | 0.765 | 154.3% |
| ***TBP***  (AALF025691) | Fw 5’GAGCAAAGCTTCGGCTACAC  Rv 5’TGAGATTCACCGTCGAAACA | 0.993 | 137.5% |
| ***α-tubulin***  2^nd^ try | Fw 5’ CGTATCATCCGTAACGGCCT  Rv 5’ GTAGAGCAGACAACAGGCCA | 0.887 | 143.2% |
| ***β-tubulin*** (AALF010418)  1^st^ try | Fw 5’ GACGAGTATCCCCGGAAGAT  Rv 5’ AGAGCTTCATTGTCCAGGCA | 0.632 | 88.7% |
| ***β-tubulin***  2^nd^ try | Fw 5’ TAGTCTCTGTGTCGGATGTAGT  Rv 5’ GACATTGTGACGGAGATCAGG | 0.911 | 122.3% |
| ***RPS7***  (AALF016123) | Fw 5’ ATGGTTTTCGGATCAAAGGT  Rv 5’ CGACCTTGTGTTCAATGGTG | 0.661 | 180.9% |
