# Supplementary material for: Assessment of Aedes albopictus reference genes for quantitative PCR at different stages of development
Source: PLoS One. 2018 Mar 19;13(3):e0194664. doi: 10.1371/journal.pone.0194664 (PMC5858815; doi:10.1371/journal.pone.0194664)
Supplement: S1 Table — A complete analysis of candidate genes performance by BestKeeper. (DOCX) [file pone.0194664.s003.docx]

| **Supplementary Table S1: BestKeeper descriptive statistic analysis** | | | | | | | | | | |
| --- | --- | --- | --- | --- | --- | --- | --- | --- | --- | --- |
|  |  |  |  |  |  |  |  |  |  |  |
| **Dev. Stage** | **Statistical Component** | ***RPS17*** | ***RPL32*** | ***eIF2α*** | ***eEF1-γ*** | ***PP2A*** | ***PGK1*** | ***ILK*** | ***STK*** | ***ACT*** |
| 0-3h | **std dev [± CP]** | **0.127** | **0.185** | **0.254** | **0.184** | **0.150** | **0.263** | **0.174** | **0.220** | **0.167** |
|  | *BestKeeper vs. coeff. of corr. ®* | 0.374 | -0.022 | 0.282 | 0.195 | 0.509 | 0.437 | 0.664 | 0.012 | 0.521 |
|  | **p-value** | 0.320 | 0.954 | 0.461 | 0.613 | 0.163 | 0.238 | 0.051 | 0.977 | 0.151 |
| 3-6h | **std dev [± CP]** | **0.070** | **0.126** | **0.099** | **0.385** | **0.489** | **0.433** | **0.425** | **0.298** | **0.553** |
|  | *BestKeeper vs. coeff. of corr. ®* | 0.145 | 0.554 | 0.782 | 0.809 | 0.839 | 0.956 | 0.965 | 0.859 | 0.701 |
|  | **p-value** | 0.708 | 0.122 | 0.013 | 0.008 | 0.005 | 0.001 | 0.001 | 0.003 | 0.035 |
| 6-9h | **std dev [± CP]** | **0.313** | **0.314** | **0.127** | **0.134** | **0.293** | **0.257** | **0.200** | **0.182** | **0.387** |
|  | *BestKeeper vs. coeff. of corr. ®* | 0.824 | 0.514 | -0.192 | 0.364 | 0.716 | 0.845 | 0.416 | -0.450 | -0.375 |
|  | **p-value** | 0.006 | 0.156 | 0.619 | 0.337 | 0.030 | 0.004 | 0.266 | 0.225 | 0.320 |
| 9-12h | **std dev [± CP]** | **0.172** | **0.236** | **0.271** | **0.655** | **0.159** | **0.170** | **0.209** | **0.164** | **0.505** |
|  | *BestKeeper vs. coeff. of corr. ®* | 0.090 | 0.104 | 0.339 | 0.440 | 0.171 | 0.367 | 0.618 | 0.494 | 0.463 |
|  | **p-value** | 0.817 | 0.788 | 0.374 | 0.235 | 0.659 | 0.333 | 0.076 | 0.177 | 0.210 |
| 12-18h | **std dev [± CP]** | **0.156** | **0.212** | **0.232** | **0.323** | **0.163** | **0.224** | **0.280** | **0.190** | **0.135** |
|  | *BestKeeper vs. coeff. of corr. ®* | -0.024 | -0.179 | 0.887 | 0.626 | 0.887 | 0.679 | 0.666 | 0.516 | 0.390 |
|  | **p-value** | 0.954 | 0.646 | 0.001 | 0.072 | 0.001 | 0.044 | 0.050 | 0.156 | 0.300 |
| 18-24h | **std dev [± CP]** | **0.201** | **0.220** | **0.161** | **0.263** | **0.263** | **0.102** | **0.300** | **0.151** | **0.236** |
|  | *BestKeeper vs. coeff. of corr. ®* | 0.264 | 0.070 | 0.353 | 0.872 | 0.210 | 0.698 | 0.592 | -0.374 | 0.771 |
|  | **p-value** | 0.495 | 0.855 | 0.351 | 0.002 | 0.586 | 0.036 | 0.094 | 0.320 | 0.015 |
| 24-48h | **std dev [± CP]** | **0.190** | **0.374** | **0.784** | **0.396** | **0.810** | **1.313** | **0.084** | **1.015** | **0.356** |
|  | *BestKeeper vs. coeff. of corr. ®* | 0.857 | -0.345 | -0.029 | 0.095 | 0.621 | 0.823 | 0.702 | 0.849 | 0.900 |
|  | **p-value** | 0.003 | 0.364 | 0.938 | 0.810 | 0.074 | 0.006 | 0.035 | 0.004 | 0.001 |
| 48-72h | **std dev [± CP]** | **0.313** | **0.394** | **0.393** | **0.758** | **1.301** | **1.319** | **1.255** | **2.467** | **0.520** |
|  | *BestKeeper vs. coeff. of corr. ®* | -0.486 | 0.745 | 0.918 | 0.006 | 0.678 | 0.935 | 0.840 | -0.774 | -0.002 |
|  | **p-value** | 0.185 | 0.021 | 0.001 | 0.985 | 0.045 | 0.001 | 0.005 | 0.014 | 0.992 |
| 1L | **std dev [± CP]** | **0.719** | **0.337** | **0.601** | **0.624** | **0.942** | **0.150** | **0.837** | **0.684** | **0.190** |
|  | *BestKeeper vs. coeff. of corr. ®* | 0.854 | -0.240 | -0.154 | 0.816 | -0.109 | -0.169 | 0.460 | -0.445 | -0.179 |
|  | **p-value** | 0.003 | 0.536 | 0.694 | 0.007 | 0.780 | 0.666 | 0.213 | 0.232 | 0.646 |
| 2L | **std dev [± CP]** | **0.947** | **0.431** | **0.830** | **0.559** | **1.367** | **1.094** | **1.981** | **1.246** | **0.840** |
|  | *BestKeeper vs. coeff. of corr. ®* | -0.332 | 0.645 | -0.051 | -0.380 | 0.301 | 0.825 | 0.831 | 0.379 | -0.814 |
|  | **p-value** | 0.383 | 0.061 | 0.893 | 0.312 | 0.429 | 0.006 | 0.005 | 0.316 | 0.008 |
| 3L | **std dev [± CP]** | **0.981** | **0.371** | **1.151** | **1.330** | **0.365** | **1.151** | **2.020** | **0.990** | **1.380** |
|  | *BestKeeper vs. coeff. of corr. ®* | 0.273 | 0.637 | -0.068 | -0.236 | -0.012 | 0.112 | 0.155 | 0.363 | -0.196 |
|  | **p-value** | 0.478 | 0.065 | 0.862 | 0.543 | 0.977 | 0.773 | 0.687 | 0.337 | 0.613 |
| 4L | **std dev [± CP]** | **0.702** | **0.166** | **1.719** | **0.518** | **0.652** | **2.102** | **1.627** | **0.300** | **0.790** |
|  | *BestKeeper vs. coeff. of corr. ®* | -0.495 | 0.547 | 0.749 | -0.796 | 0.959 | 0.741 | 0.233 | 0.233 | 0.291 |
|  | **p-value** | 0.175 | 0.127 | 0.020 | 0.010 | 0.001 | 0.022 | 0.549 | 0.549 | 0.450 |
| Pupae | **std dev [± CP]** | **0.633** | **0.362** | **2.193** | **1.493** | **0.987** | **1.351** | **2.638** | **0.169** | **0.920** |
|  | *BestKeeper vs. coeff. of corr. ®* | 0.372 | 0.724 | 0.930 | 0.369 | 0.936 | -0.394 | 0.938 | -0.675 | -0.510 |
|  | **p-value** | 0.324 | 0.027 | 0.001 | 0.329 | 0.001 | 0.296 | 0.001 | 0.046 | 0.160 |
| Adult, Male | **std dev [± CP]** | **0.336** | **0.901** | **0.264** | **0.246** | **0.801** | **0.259** | **0.937** | **0.511** | **0.320** |
|  | *BestKeeper vs. coeff. of corr. ®* | -0.289 | -0.182 | -0.035 | 0.340 | 0.882 | 0.321 | 0.352 | 0.798 | -0.088 |
|  | **p-value** | 0.450 | 0.639 | 0.931 | 0.369 | 0.002 | 0.398 | 0.351 | 0.010 | 0.825 |
| Adult, Female | **std dev [± CP]** | **0.728** | **0.847** | **0.268** | **0.598** | **0.663** | **0.421** | **0.338** | **0.389** | **0.960** |
|  | *BestKeeper vs. coeff. of corr. ®* | 0.960 | 0.934 | -0.140 | 0.956 | 0.322 | 0.819 | -0.025 | -0.437 | 0.873 |
|  | **p-value** | 0.001 | 0.001 | 0.722 | 0.001 | 0.398 | 0.007 | 0.946 | 0.238 | 0.002 |
| Adult, Female, 24h PBM | **std dev [± CP]** | **0.320** | **0.335** | **1.058** | **0.615** | **1.059** | **1.454** | **1.013** | **0.927** | **0.510** |
|  | *BestKeeper vs. coeff. of corr. ®* | 0.843 | 0.864 | 0.995 | 0.938 | 0.946 | 0.986 | 0.916 | 0.914 | 0.953 |
|  | **p-value** | 0.004 | 0.003 | 0.001 | 0.001 | 0.001 | 0.001 | 0.001 | 0.001 | 0.001 |
| Cells | **std dev [± CP]** | **0.764** | **0.350** | **0.952** | **0.303** | **0.386** | **1.630** | **0.295** | **0.639** | **1.490** |
|  | *BestKeeper vs. coeff. of corr. ®* | 0.716 | 0.842 | 0.976 | 0.899 | -0.669 | 0.976 | 0.270 | -0.913 | 0.968 |
|  | **p-value** | 0.030 | 0.004 | 0.001 | 0.001 | 0.049 | 0.001 | 0.483 | 0.001 | 0.001 |

# **Supplementary Table 1.** A complete analysis of candidate genes performance by BestKeeper. Boxes highlighted red indicate instances where the candidate genes exhibited a standard deviation greater than 1; BestKeeper recommends the exclusion of the gene from further analysis in such cases, as this typically indicates its instability within the sample type. For *BestKeeper vs. coeff. of corr.*, the closer the value is to 1, the greater the gene’s ability to normalize gene expression for the sample type.
